# Improving employment retention at a North West province nursing college: Perceptions of nurse educators

**DOI:** 10.4102/hsag.v31i0.3278

**Published:** 2026-04-28

**Authors:** Ernest H. Mbolongwe, Emmerentia du Plessis, Verena L. Neethling

**Affiliations:** 1 Department of Quality Nursing and Midwifery (NuMIQ), Faculty of Health Sciences, North-West University, Potchefstroom, South Africa

**Keywords:** nursing education, nurse educators, public nursing college, retention, retention strategies, staff turnover, workplace perceptions

## Abstract

**Background:**

High nurse educator turnover rates necessitate the need for nursing colleges to explore the retention of nurse educators. The views of nurse educators were sought to generate contextually informed insight into improving retention.

**Aim:**

To explore and describe the perceptions of nurse educators working at a public nursing college in North West, South Africa, on improving retention of nurse educators.

**Setting:**

The research was conducted at two campuses of a public nursing college in the North West province.

**Methods:**

A qualitative descriptive design was used to explore and describe the perceptions of nurse educators in providing answers to the research question. Typical purposive sampling was used by applying selection criteria. Focus group interviews were conducted, and conventional content analysis was applied. Data saturation was reached after conducting four focus group interviews, with a total of 29 participants, divided as follows: Campus A: two groups of eight participants each, Campus B: one group with six participants and one group with seven participants.

**Results:**

Five categories with sub-categories emerged, namely: (1) improving the working environment contributes towards retention, (2) improving human resource management, (3) better remuneration and benefits, (4) positive aspects of staying in nursing education and (5) eliminating politics and mismanagement.

**Conclusion:**

This study on the views of the nurse educators acknowledges their voices, ensuring that the matter of retention can be informed by those directly situated within the phenomenon.

**Contribution:**

The study provides insight into the views of nurse educators regarding employment retention, namely that the work environment – including the physical environment, human resource management, remuneration and benefits and political influence and mismanagement – as well as nurse educators’ passion for what they do, influences their decision to leave or to stay in employment. These insights are valuable for tailoring retention strategies and provide a solid grounding for further research on the employment retention of nurse educators.

## Introduction

Improving employment retention is a phenomenon affecting public nursing colleges both nationally and globally, including a public nursing college in the North West Province of South Africa. Boamah et al. (2023:8) confirm that the absence of job satisfaction, lack of recognition and insufficient remuneration often lead nurse educators to leave their employment. It is recommended that nursing colleges and health department authorities implement measures and strategies as suggested by nurse educators themselves in improving retention so that work overload resulting from these shortages will not be experienced (DeWitty & Murray 2020:42).

There is a need to explore and describe the perceptions of nurse educators on retention at public nursing colleges. Frost (2022) agrees that ways must be devised to attract and retain nurse educators in public nursing colleges. Research is thus needed to address staff shortages, which contribute to work overload and burnout among the remaining nurse educators (Christian 2021; Rikhotso 2018).

This study originated from the observations made by the first author while working as a public nursing college nurse educator, who experienced the intensity of the resignations of colleagues leaving for other (perceived to be) more attractive employment opportunities. This attraction involved better remuneration, professional respect and conduct. Rikhotso (2018) confirms this observation regarding South African public nursing colleges, while Christian (2021) argues that the retention of nurse educators in nursing colleges warrants exploration on a global scale.

This study at a public nursing college adds to what has already been written on the subject, and is in line with literature that emphasises the need for college authorities to urgently attend to the matter of retention (Ariana, Soleimane & Oghazian 2018).

The purpose of the study was to explore educators’ perceptions on improving retention at a public nursing college in the North West province of South Africa.

## Research design and method

A qualitative descriptive design (Sandelowski 2000:334) was used to gain in-depth information by encouraging the participants to provide their perceptions on improving retention at the college. This design paved the way for an in-depth exploration and description of the underlying views and reasoning of the participants, gaining rich insights that can contribute to the design of relevant strategies (Busetto, Wolfgang & Gumbinger 2020:1).

### Setting

The setting of the study was that of a public nursing college in the North West. The campuses offer nursing education programmes prescribed by the South African Nursing Council, referred to as the Regulation 171 programme and the Regulation 425 programme (the latter being phased out). The public college operates 5 days a week, and working hours are between 07:00 and 16:00. The data collection was done within these working hours with no disruption of the participants’ main responsibilities. Data collection was done in closed, well-lit and carefully arranged boardrooms with ‘do not disturb’ signs on the doors.

### Study population and sampling strategy

The population included all nurse educators who had been employed at the college for 1 year or longer.

Campus A’s population was 34, while the population at Campus B was 45 (thus a total population of 79).

Typical purposive sampling was applied (Nyimbili & Nyimbili 2024:95). Inclusion criteria entailed that nurse educators who were employed at the selected Campus A and Campus B of a public nursing college in North West were eligible to participate, based on the reasoning that they were able to provide detailed information.

Nurse educators who have been employed for less than a year at the nursing college were excluded. Participants were recruited with the assistance of a mediator at each campus, who informed potential participants about the research, using word of mouth and pamphlets. Potential participants were invited to inform the mediator if they were interested in participating in the research, after which written informed consent was obtained.

Campus A had 16 participants, divided into two focus groups of eight in each group. At Campus B, participants were also divided into two focus groups, with seven participants and six participants in the groups, respectively.

### Data collection

After obtaining all the permissions from the public college in North West and the Department of Health, the data collection was done during August and September 2024. Four face-to-face focus group interviews with semi-structured questions, as described by Krueger and Casey (2014), were held.

The first author facilitated the semi-structured focus group interviews after participants voluntarily signed informed consent forms. Only the first author and the participants were present during the focus group interviews. The first focus group interview was seen as a pilot interview. No changes to the interview questions were necessary. The data from this interview could be included in the data set. The interview questions were semi-structured and open-ended, and were: What motivates you to stay employed at the nursing college? What do you consider the main reasons why you would leave your current employment at the nursing college? What would bring more fulfilment in your nurse educator job that will help you to stay here at the nursing college? What suggestions do you have regarding retaining nurse educators at this nursing college?

The focus group interviews were audio-recorded on two separate recording devices. Data saturation, namely that no new data, meaning and themes emerged, was reached at the fourth focus group interview, with a total of 29 participants for the study. The focus group interviews lasted between 45 and 90 min each, and field notes were taken directly after each focus group interview. These field notes were read in conjunction with the transcripts of the interviews to enrich data analysis. The field notes included notes on the setting, participants’ responses and own reflections (Phillippi & Lauderdale 2018:385).

### Data analysis

Data was analysed using conventional content analysis, namely that codes are derived from the data (Hsieh & Shannon 2005:1279). The first step was listening to the recordings, followed by transcribing and refining the transcriptions for better coding and interpretations. The transcripts were analysed manually.

The transcripts were read repeatedly to become familiar with the tacit meaning and content of the data sets (Bryman 2016). Thereafter, codes were identified from the text by highlighting meaningful words and phrases. The codes were related and linked based on similarities, differences and nuances within and across transcripts (Hsieh & Shannon 2005:1279). Similar codes were combined to form categories and sub-categories (Hsieh & Shannon 2005:1279). A co-coder was involved in data analysis to ensure trustworthiness and to exclude possible researcher biases (Hsieh & Shannon 2005:1280).

The first author and co-coder analysed the data separately before meeting to discuss the codes generated and reach consensus on the categories and sub-categories.

### Measures of trustworthiness

According to Conelly (2016:435), measures of trustworthiness in qualitative research are measures to ensure the truthfulness, authenticity and quality of the research findings.

Credibility ensured that the study’s findings were grounded in the data and reflected participants’ perceptions (Cypress 2017:253). Credibility strategies were applied by ensuring that the participants were thoroughly informed about the research and of what would be expected of them through obtaining written, voluntary, informed consent, time spent holding the interviews, adequate time describing the study and the briefing of the participants.

Furthermore, researcher reflexivity was applied through taking field notes and through regular reflexive meetings among the research team to ensure awareness of and limiting preconceptions, following a data analysis method that is aligned with a descriptive qualitative design, namely to stay close to the accounts of the participants and to present the findings as close as possible to the participants’ language and meaning (Sandelowski 2000).

Transferability (applicability) was ensured through providing a thick description of the background, context and research methodology of the study, enabling researchers, policy makers and nurse educators to review the findings of the research as transferable or applicable in other contexts. Dependability (consistency) was established, also through the thick description of the research, ensuring that other researchers could track the precise research method in a way that the study could be replicated to generate similar results. Confirmability (neutrality) was established, using the strategies mentioned above, to ensure that the research findings are based on the narratives of the participants and not invented by the researchers. The researchers used all the mentioned strategies to authenticate the findings.

### Ethical considerations

Approval was obtained for the study from the North-West University Health Research Ethics Committee (NWU-HREC). The ethical clearance number is NWU-00043-21-A1. Risks from the study were minimal, and the research did not raise any ethical issues. No other intentions or personal interests formed any biases in the study. Written and signed informed consent documents were obtained from the participants, who were also informed of their rights before data collection. The participants were informed that they were free to withdraw from the study at any time. Privacy was ensured through conducting the focus group interviews in private rooms, with no disturbances. Confidentiality and anonymity of the data were ensured through not sharing the data with any person outside the research team, removing identifying information from the transcripts and storing the data securely.

## Findings

The findings of the study include an overview of the demographic data of the participants and a presentation of the five categories (with sub-categories) that resulted from data analysis. The demographic data of the participants are presented in Table 1. This data includes participants from both campuses, detailing their gender, age, years of work experience and the number of participants from each campus.

**TABLE 1:** Demographic information of the participants.

Campus and/or focus group	Number of male participants	Number of female participants	Ages in years	Total number of participants in focus group interviews	Years of experience as a nurse educator
**Campus A**					
Focus Group 1	0	08	28–65	08	3–35
Focus Group 2	01	07	32–58	08	1–42
**Campus B**					
Focus Group 3	01	06	26–54	07	5–36
Focus Group 4	0	06	33–61	06	2–44

Table 2 shows all the categories that naturally flowed from the research information gathered. The categories and sub-categories are presented through a short description supported by quotations from the participants, and pseudonyms (Participant A, B, C, etc.) are used to refer to each participant.

**TABLE 2:** Categories and sub-categories.

Category	Sub-category
1. Improving the working environment contributes towards retention	1.1 Environment more conducive 1.2 Need for resources 1.3 Recognition and succession 1.4 Need for continuous development
2. Improving human resource management	2.1 Role ambiguity 2.2 High workload 2.3 Understaffed
3. Better remuneration and benefits	3.1 Competitive and equitable salaries 3.2 Comprehensive employment benefits
4. Positive aspects of staying in nursing education	4.1 Passion for nursing 4.2 Better working hours
5. Eliminating politics and mismanagement	5.1 Need for less political interference 5.2 Need for improved management

### Category 1: Improving the working environment contributes towards retention

Participants shared that improving the work environment contributes towards retention, as described in the following sub-categories.

#### Sub-category 1.1: Environment more conducive

The participants expressed concern and frustration that the ongoing construction at both campuses interfered with their work and posed a safety risk to them and their students. They expressed the need for a safe environment, free from the constant noise of hammering and drilling, and the unsafe circumstances of building materials and incomplete walls with running electricity cables on the floors.

Their frustration and concern were evident from the following quotes:

‘This college, to be honest, is not complete; this college is being built for six years, up to now, it’s not even complete.’ (Participant C, Campus A, Focus Group 1, 7 years work experience)

‘And the other thing is, this college has been under construction for, I don’t know, for how many years. I’m going back to the safety issue. At times, the construction, the grinders will be grinding while we are busy in our offices and classes, yet we are not allowed to be here at the building.’ (Participant F, Campus B, Focus Group 4, 15 years work experience)

The participants explained that the prolonged renovations are not only a safety risk and an interference, but to them it is also a sign of not being heard by their managements:

‘I am of the view that the college should take our needs seriously because I have the power to do something about it, and in this case, I can share the state of the college. It’s been a decade or more now that this project is not finished. So, it will be very fulfilling to all of us if we can have a proper functioning college.’ (Participant F, Campus A, Focus Group 1, 16 years work experience)

#### Sub-category 1.2: Need for resources

Another major challenge identified by the participants in the public nursing college is a lack of resources, such as not having safe and reliable transport to do clinical accompaniment and access to updated teaching material.

The participants’ demotivation because of not having enough resources is evident from the following quote:

‘I think like we all being totally overwhelmed by not having resources. Talking about [the need for] preceptors and we are taking our own cars to go and assess students. I had to take my laptop last week to the computer shop to update Microsoft because my videos didn’t even want to show in class.’ (Participant H, Focus group 1, Campus A, 8 years work experience)

The participants’ frustration deepened because resources are managed externally, by persons who do not prioritise the college and who are not in touch with the needs of the nurse educators, as evident from the following quotes:

‘Politicians who are running the show politically and then you will find out that shortage of resources is also the fact that the politicians are the ones who are determining the budget for the college, the budget for the hospitals, and now you find out that they give first priority to these games, because their hospitals, the clinics and college, are forgotten.’ (Participant D, Campus A, Focus group 2, 19 years work experience)

‘The people that are managing the department should be people that know what the needs at the grassroot are, that’s why we need this because now the reason for us to suffer is because really there are people who are politically interfering, being people who don’t even know what we are doing.’ (Participant B, Campus B, Focus group 4, 2 years work experience)

#### Sub-category 1.3: Recognition and succession

The participants viewed the lack of recognition and succession opportunities in the public nursing colleges as extremely demotivating. Prior work experience is not recognised for remuneration increases, promotion considerations or performance bonuses. Furthermore, additional postgraduate qualifications are not taken into consideration when increasing salaries, promotions or other opportunities in nurse education.

The following quote illustrates the participants’ feeling of demotivation regarding not being recognised for their prior work experience and post-graduate qualifications:

‘Oh, let’s see. One of the reasons why I would leave is. I don’t know how to put it, but failure of the government or the college to recognise qualifications, you basically have to wait for a post in order for you to be given a better salary. You can have a master’s degree for five years as a lecturer, but you will not get anything, any recognition. I mean recognition in many ways.’ (Participant A, Focus Group 2, Campus A, 11 years work experience)

Participants also experienced that succession planning is very limited and unfair because of the few promotion opportunities that arise, while at the same time, they are expected to act on behalf of their line managers on short notice, as evident from the following quote:

‘No, I was just saying that there must be a succession plan for us as lecturers, because there is no succession plan when the HOD is not around, then you are told to act, just grabbed to go and act. You don’t know what is happening there. You are just needed to act and now to learn. Then you have no opportunity to get that position unless you really get luck and the chances are almost zero.’ (Participant E, Focus Group 3, Campus B, 3 years work experience)

#### Sub-category 1.4: Need for continuous development

The participants believed that continuous development opportunities would be a valuable way to retain nurse educators at the college. At the same time, they perceived that there was a lack of development opportunities at their workplace. There is also a perception that there is favouritism, namely that not all nurse educators have an equal opportunity to be sent for further training.

The participants emphasised the importance of development while being employed at the college, as seen in the following quote:

‘Yes, to add on this, to retain people, people must be allowed to go for development. Continuous education is very, very important. People must be allowed, given time to go and develop themselves from the very college.’ (Participant F, Focus Group 3, Campus B, 16 years work experience)

Their frustration with favouritism regarding continuous development opportunities can be seen from the following quote:

‘I perceive favouritism at work in the college. This is because I personally am still waiting for further studies development for over seven years now, it has not happened. A more newly appointed lecturer with only five years of service who is very close in relationship with one of the campus management personnel has just been approved to go for further studies on a provided bursary.’ (Participant C, Focus group 2, Campus B, 9 years work experience)

### Category 2: Improving human resource management

The participants indicated that improved human resource management will contribute to retention. They specifically expressed that their roles are constantly changed to cover for higher workloads and understaffing. This situation causes discontent. The sub-categories below address this view.

#### Sub-category 2.1: Role ambiguity

The participants perceived role ambiguity at the college, making their work unpleasant. They shared that new roles are allocated on short notice, and it takes time to explore the newly allocated role. While still struggling to learn the role, another role is assigned, usually unrelated to the current one. Participants indicated that it was their expectation to continue with the current role till they have grasped it, before being given a new one.

The following quote indicates their discouragement:

‘Yeah, still with the allocation really, to me, allocation is not nice. You will be doing an important administration task in your office, and the due date is here to submit, then someone comes, takes you out of that, come and help here, you leave this one, as if it is not important.’ (Participant E, Focus Group 2, Campus A, 26 years work experience)

Participants found it difficult to cope with their duties in such circumstances, and they felt their autonomy was not respected:

‘I find it difficult to cope effectively with my duties because while I am busy preparing students for theory content, I am called to leave them and sent to the hospital to accompany other students and provide assessment preparations.’ (Participant D, Focus Group 1, Campus A, 6 years work experience)

‘We are usually not given a choice on what subject allocation I would wish to belong to. This deprives me [of] autonomy to choose a subject I will be most effective in, especially when I don’t yet have many years of teaching experience. No proper induction is given and that sometimes shows incompetence before the students.’ (Participant B, Focus Group 2, Campus A, 4 years work experience)

#### Sub-category 2.2: High workload

The participants viewed work overload as a basis for burnout because of the nurse educator shortages that caused an overburden of responsibilities. The participants shared that they had to do the work of two to three people and do excessive administrative work in addition to their nursing education duties.

They perceived a high workload as one of the reasons to resign, because it places additional pressure on them and overburdens them, as seen in the following quote:

‘Unfortunately, work overload persists because some lecturers resign, no one is immediately put to replace him or her, and the work is then shared between the already overburdened nurse educators that remain. This becomes a crucial reason to overwork the ones that remain.’ (Participant G, Focus Group 2, Campus A, 7 years work experience)

The participants made suggestions on how to cope with the high workload, such as each one taking full responsibility for their own role, and having temporary lecturers available to help relieve the workload:

‘Ya, to add on that, the workload will be resolved if each and every one in their own space do their work. Trying to make sure that we submit things in time, we meet deadlines, every one of us needs to be be correctly allocated.’ (Participant B, Focus Group 1, Campus A, 6 years work experience)

‘There is need for standby lecturers to come in and assist when there are shortages such as the ones that we experience, even on an ad hoc basis. This would minimise work overload.’ (Participant E, Focus Group 1, Campus A, 13 years work experience)

#### Sub-category 2.3: Understaffed

The participating nurse educators identified understaffing as another reason to leave the nursing colleges, and that it takes a long time before replacements are made. This usually makes the remaining nurse educators face high workloads, and this also causes a reduction in the student intake number, thus reducing the number of nurses of all categories being trained for service.

The following quote illustrates that because of being understaffed, the participants experienced work as exhausting and that time management was impossible, impacting their work-life balance:

‘Too much workload, I don’t separate administration work and the teaching work, so you do all the things at your own time, and even when you are off duty, you still continue with work at home, and no one is appreciating it.’ (Participant G, Focus Group 1, Campus A, 19 years work experience)

Furthermore, the participants shared that because of being understaffed at the college, they are expected to fulfil the role of lecturer, administrator and preceptor, which in their view is not the case at other educational institutions, such as universities. They experience this as unfair and overwhelming:

‘At universities, they have preceptors who assist with the workload regarding clinical accompaniments, thus normalising the work of the other lecturers. It is not happening here. We lecture and prepare assessments and give to students and mark their assignments and written assessments. We then turn to the accompaniment of students in teaching and assess procedures that we taught them, preparing them for OSCE, it’s too much.’ (Participant A, Focus Group 3, Campus B, 15 years work experience)

### Category 3: Better remuneration and benefits

Nurse educators from all four focus group interviews perceived better remuneration and benefits as vital in improving employment retention. This view is described in the following sub-categories.

#### Sub-category 3.1: Competitive and equitable salaries

Participants’ perceptions were that their salary bracket is not competitive and equitable, and they are dissatisfied that it remains at the same level for as long as 10 years before it is changed to the next level. Participants expressed unhappiness regarding this matter, and they noted their readiness to leave this employment if a better salary is offered elsewhere.

The participants elaborated on this viewpoint, explaining that their remuneration packages do not include the same incentives as for nurses working in a speciality post in clinical practice:

‘In terms of salary, the starting salary for lecturers is the same as the clinical hospital nurse who comes with the same speciality. You have the overtime, also, even some sort of development is there, but here you are not developed, the salary doesn’t have the incentives that you normally get in the clinical area, so it’s demoralising.’ (Participant B, Focus Group 4, Campus B, 23 years work experience)

They perceived this situation as discouraging, as their expertise is not recognised, as expressed in the following quote:

‘Another thing is that they are just focusing on nursing education. What about your other nursing experience? 30 years’ experience in nursing and that has not been acknowledged! You see and that is the killer, that is what is killing us as nurse educators.’ (Participant A, Focus Group 1, Campus A, 34 years work experience)

The participants were adamant that being a nurse educator is a high-level post, which is not acknowledged in their remuneration packages. This leads to resignations, as seen in the following quote:

‘This is because nurse educators’ perceptions of their work are equal to that of an operation manager in a hospital or clinic or an assistant nursing manager or even a nursing manager. Most nurse educators have management qualifications and opt to leave for those better paying positions.’ (Participant D, Focus Group 2, Campus A, 17 years work experience)

#### Sub-category 3.2: Comprehensive employment benefits

The participants expressed dissatisfaction with the lack of benefits or incentives to motivate them in their work. They were of the view that they had been reduced to just workers who must meet targets set for them. They also recalled that they would appreciate recognition in the form of award ceremonies and tokens of appreciation.

The following quote supports this finding and illustrates that the lack of incentives, in this case, limited opportunity for professional development, is one of the reasons that can lead to nurse educators leaving their positions:

‘What will make me to leave my current employment is lack of professional development. As a nurse educator, you can only move up if you become an HOD. No development or financial benefits are available, and for you to be an HOD you must study further and compete with over 30 other colleagues wanting the same position.’ (Participant B, Focus Group 4, Campus B, 12 years work experience)

The participants expressed the need for acknowledgement, which they perceive will be motivating. The following quotes illustrate this finding:

‘I think as well, we should have gala dinners, we should have awards, even if you did not do something big, just this nice ticket of acknowledgement to be told that we thank you for your work. An appreciation that this time you worked so hard though you did not get 100% pass rate. Acknowledgement also that you put in work and effort for those students will fulfil you to say, I can go back to work tomorrow!’ (Participant E, Focus Group 4, Campus B, 6 years work experience)

‘Oh yes, certain incentives must be devised to acknowledge all the hard work nurse educators put in their work such as shopping vouchers or even bus tickets or air tickets to holiday destinations, well planned to motivate the nurse educators.’ (Participant G, Focus Group 1, Campus A, 11 years work experience)

### Category 4: Positive aspects of staying in nursing education

The participants acknowledged and explored several positive aspects of remaining in nursing education. This includes their passion for the field and the fulfilment they experience in seeing their students graduate and enter the nursing profession. They also appreciated the flexible working hours, time off on weekends and holidays, as well as recess periods.

#### Sub-category 4.1: Passion for nursing

The nurse educator participants alluded to the passion for nursing education as a motivational factor that made them stay employed. They experienced satisfaction in seeing their students working in practice and providing nursing care.

Specifically, the participants felt motivated by being able to share their skills with future generations of nurses, contributing to improved professionalism in nursing care. The following quotes describe this motivation:

‘Actually, I can just say this, something that motivates me to stay here is the love I have for nursing education, but most importantly, to be able to share my skills with the upcoming nurses.’ (Participant H, Focus Group 1, Campus A, 14 years work experience)

‘I am motivated to stay at the nursing college because, generally, nursing is my passion and if we are aware now of late, people are not looking at us so good because of the type of nurses that we are producing. So, I decided and concluded to stay because I know I’ve realised that I’m having that potential to at least instil the culture of professionalism and ethics in nursing.’ (Participant F, Focus Group 3, Campus B, 7 years work experience)

The participants were also motivated through the gratitude expressed by students:

‘My motivation is that I get fulfilled to see my student products serving clients diligently and receiving gratitude from the students that they are better at their work because they received good training. The appreciation that they show is so fulfilling.’ (Participant G, Focus Group 1, Campus A, 14 years work experience)

#### Sub-category 4.2: Better working hours

The participants shared that better working hours than in clinical nursing practice motivated them to stay in their nursing education position, namely, having weekends and holidays (periods when students are also on recess) off.

In contrast to what the participants shared earlier regarding the difficulties they experienced in time management, they also expressed their appreciation for the flexibility in their schedules and not having to work on weekends and recess times, as compared to nurses working in clinical practice:

‘The other motivation is that on weekends we are always off as well as on public holidays and when students are on recess.’ (Participant E, Focus Group 2, Campus A, 19 years work experience)

‘Even when at work, we go to class or for accompaniment at allocated times, giving us flexibility to knock off when there is still enough time to get to our homes not too late and arriving for work not too early in the morning.’ (Participant F, Focus Group 4, Campus B, 15 years work experience)

They furthermore expressed a positive view on having enough time to spend with students, because of the flexibility in their schedules:

‘We are also able to spend enough time with our students both at the college for the theory component and at the clinical facilities. This improves our student pass rate because we prepare well due to the abundance of flexible time, we sometimes get to be with them.’ (Participant G, Focus Group 2, Campus A, 6 years work experience)

### Category 5: Eliminating politics and mismanagement

Participants expressed concern and dissatisfaction with perceived political interference and mismanagement regarding the allocation of resources and appointments. This view is described in the following sub-categories.

#### Sub-category 5.1: Need for less political interference

The participants perceived that officials within the provincial health department and health governance structures influence decision-making processes at the college level, even though they usually did not understand the needs of the colleges and the nurse educators, as they would typically not have a healthcare or nursing background. The participants further perceived the college to be dependent on provincially appointed managerial figures who are involved in oversight, budgeting and strategic decision-making at a provincial level. This perceived external political influence was associated with a continual lack of essential resources and equipment, which participants experienced as limitations in autonomy and decision-making authority within the college.

The participants viewed this trend as discouraging, as demonstrated by the following quote:

‘The current dispensation whereby nursing is no more controlled by nurses. We are reporting to somebody else who is not a nurse and does not understand the dynamics of nursing. That is why, eventually, then, the lack of resources, because the rule should be that of understanding why you need it. Why do you say you want this type of a resource? As a result, you become discouraged. You don’t have your own innovations or ideas to order resources. Somebody else must give it out, who is not a nurse but a politician.’ (Participant D, Focus Group 2, Campus A, 21 years work experience)

The participants were very concerned about how the control of finances by politicians causes shortages in important resources, as seen in the following quote:

‘I fully agree to the fact that politicians in control of finances in nursing usually look for political good names by reducing most needed equipment and other resources. This is because they want to be praised by returning money allocated for the resources to treasury so that they are seen to be good at spending public funds. This brings in suffering on the nurse educators who are perpetually experiencing shortages of everything including unserviced cars and other equipment.’ (Participant D, Focus Group 3, Campus B, 21 years work experience)

#### Sub-category 5.2: Need for improved management

The participants expressed the need for improved management to improve the retention of employees. Management was perceived as falling short of the required standard of treating nurse educators as valued and respected employees.

The following quote is evidence of the participants’ need to be treated with respect by their managers:

‘You know, they need to report bullying, coming to an office, before even saying anything, you are met with questions like, “who told you to come in?.” When you go in, you are asked, “what are you doing here? I didn’t say come in.” So, I think we also need to treat each other with respect.’ (Participant B, Focus Group 3, Campus B, 6 years work experience)

In addition, the participants expressed concern about unfair treatment that makes them feel unwelcome, as seen in the following quote:

‘Management is not treating us equally. There are those who are sweethearts of our campus managers so to speak, and those of us who you see and experience the dislike. There is no prior fight or disagreement, but you just really see that I am not welcome here.’ (Participant D, Focus Group 4, Campus B, 16 years work experience)

## Discussion

The study’s key findings provide a clear indication of nurse educators’ perceptions of employment retention at a public nursing college in South Africa. These findings are supported by relevant literature, as discussed below.

Firstly, nurse educators perceive that an improved working environment, including working in a safe, conducive environment and access to resources, will improve retention. This finding is supported by Mthimunye and Daniels (2019), who reported that the physical work environment plays a significant role in job satisfaction, as it influences the extent to which nurse educators can fulfil their roles and create a conducive learning environment for students.

Furthermore, the finding that nurse educators viewed the lack of recognition, succession opportunities and continuous development as demotivating is echoed in the work of Matahela (2025:241), who argues that the need for recognition is a deeply rooted human need, which can be demonstrated through transparency and fairness in promotions and forms of recognition and through offering opportunities for professional development.

Secondly, the participants perceived that improving human resource management is crucial for retention. Participants shared that role ambiguity causes discontent, makes their work unpleasant and makes them feel disrespected. Tufano, Summers and Covington (2023:3) confirm that role transition can be experienced as overwhelming, leading to role strain and job satisfaction, which are key determinants in retention. Moreover, participants perceived a high workload and being understaffed as unfair, leading to feeling overwhelmed and exhausted.

Relevant literature confirms that poor human resource management leads to resignations and highlights the need to invest in the well-being of nurse educators by addressing insufficient resources and the shortage of staff (DeWitty & Murray 2020).

Thirdly, the study found that there is a need for better remuneration and benefits, specifically the need for competitive and equitable salaries and comprehensive employment benefits as a recognition of expertise and appreciation. Rikhotso (2018) recorded that public nursing colleges must consider salaries and benefits that are fair to the work and qualifications of nurse educators. Keener et al. (2021) confirm that adequate salaries and benefits are major motivators. If these conditions are met, they can arouse positive feelings and a desire to excel.

Fourthly, participants shared that the positive aspects of being a nurse educator motivated them to remain in their posts. These positive aspects include their passion for nursing education, the fulfilment they experience when nursing students complete their studies and start their nursing career, and the flexible work schedule.

This finding is strongly echoed in the literature. Tufano et al. (2023:1) also found that nurse educators’ love for teaching and satisfaction from their professional role are major motivators to stay committed and to adjust despite the challenges of the work environment, and they refer to these motivators as intrinsic motivators.

Lastly, the participants expressed the need for less political interference and improved management. They emphasised that their autonomy in decisions on resources and appointments is not recognised, and that they feel disrespected and not valued. Unfortunately, this dilemma is confirmed in similar research that describes that nurse educators at public nursing colleges experience management to be ineffective in providing strong leadership that helps them cope with the demanding nursing education environment (Ndawo 2022:9).

The study is limited to one public nursing college in South Africa, and to exploring and describing the perceptions of nurse educators. However, a thick description of the setting, method and findings is provided, enabling transferability of the findings. Future research will benefit from involving members of the management of public nursing colleges, as well as nursing students, to ensure a more comprehensive description of the phenomenon.

The implications of this research are that a deeper insight is gained into the retention of nurse educators. This insight offers guidance for nurse educators, management of public nursing colleges and researchers on the retention of nurse educators.

## Conclusion

The study explored and described the perceptions of nurse educators in a public nursing college regarding what would improve their employment retention in the college. The findings revealed that retention of nurse educators relates to the work environment, human resource management, remuneration and benefits, and the management of the college, with an underlying theme of the need for autonomy and the need to be recognised, respected and valued. In addition, nurse educators’ passion for their work motivated them to resiliently remain in employment.

Furthermore, this study offers evidence of the value of consulting those directly situated within the phenomenon, in this case, the perceptions of nurse educators on retention.
